# An inhibitor of 11-β hydroxysteroid dehydrogenase type 1 (PF915275) alleviates nonylphenol-induced hyperadrenalism and adiposity in rat and human cells

**DOI:** 10.1186/s40360-018-0235-0

**Published:** 2018-07-18

**Authors:** Ling-Ling Chang, Wan-Song Alfred Wun, Paulus S. Wang

**Affiliations:** 1 Department of Chemical and Materials Engineering, Chinese Culture University, Shih-Lin, Taipei, 11114 Taiwan Republic of China; 2 Fertility Specialists of Houston, Houston, TX 77054 USA; 3 Department of Physiology, School of Medicine, National Yang-Ming University, Taipei, 11221 Taiwan Republic of China; 4 Department of Medical Research and Education, Taipei Veterans General Hospital, Taipei, 11217 Taiwan Republic of China; 5 Medical Center of Aging Research, China Medical University Hospital, Taichung, 40402 Taiwan Republic of China; 6 Department of Biotechnology, Asia University, Taichung, 41354 Taiwan Republic of China

**Keywords:** NP, PF915275, 11β-HSD1, Hyperadrenalism, Adipogenesis

## Abstract

**Background:**

Nonylphenol (NP) is an environmental endocrine-disrupting chemical (EDC) detected in human cord blood and milk. NP exposure in developmental periods results in hyperadrenalism and increasing 11β-hydroxysteroid dehydrogenase I (11β-HSD1) activity in an adult rat model. Alleviating 11β-HSD1 activity is therefore a logical and common way to treat hyperadrenalism. PF915275 (PF; 4′-cyano-biphenyl-4-sulfonic acid (6-amino-pyridin-2-yl)-amide) is a selective inhibitor for 11β-HSD1. This study aimed to determine whether PF915275 could alleviate the hyperadrenalism induced by NP. In addition to a rat model, the effects of NP and PF915275 were measured in human preadipocytes.

**Methods:**

For the in vivo rat model, female adult rats exposed to NP during the developmental period were divided into two treatment groups, with one receiving oral DMSO solution and the other receiving PF915275 once per day for 4 weeks. After the final treatment, the rats from each group were sacrificed for analysis. For the in vitro human model, human preadipocytes received 2 regimens of NP treatment. One treatment regimen occurred before differentiation (to mimic the sensitive developmental period; P exposure), and the other included continuous exposure from preadipocytes to fully differentiated adipocytes (to mimic the growing and adult periods, respectively; C exposure). Protein and RNA were extracted from rat tissues and the preadipocytes for western blot and real-time PCR analysis.

**Results:**

In the rat model, PF915275 alleviated NP-induced effects by interfering with adipogenesis pathways, including enhancing PPARα expression, decreasing PPARγ expression, and reducing both 11β-HSD1 protein and mRNA expression levels. Additionally, PF915275 reduced the effects of the adrenal corticoid synthesis pathway by reducing StAR expression and 11β-hydroxylase and aldosterone synthase activities. With short-term exposure, NP enhanced *PPARγ* and *FASN* mRNA expression levels and reduced PPARα expression, whereas PF915275 alleviated these effects. With C exposure, the NP-induced accumulation of intracellular lipids was reduced by PF915275 treatment, which was mediated by decreased PPARγ mRNA and protein expression levels and increased PPARα protein expression.

**Conclusions:**

The effects of NP and PF915275 treatment in both rat and human cell models are similar. Rats may be an appropriate model to study the effects of NP in humans, especially during the developmental period.

## Background

Man-made chemicals, such as bisphenol A (BPA), dichlorodiphenyl-trichloroethane (DDT), diethylstilbestrol (DES), and nonylphenol (NP), are produced with the intention of benefiting humans. However, these chemicals can also have negative impacts on the environment, wildlife, and public health. These chemicals are categorized as endocrine disruptors (ENDRs) because they mainly target endocrine pathways (i.e., estrogen or corticoids) and can interfere or disrupt physiological endocrine regulation. The incidence of metabolic syndromes has been suggested to correspond with the number of synthetic chemicals produced, especially ENDRs [1]. The pandemic metabolic syndromes, such as obesity, diabetes, and hypertension, are global public health issues [2, 3]. Experts predict that the burden of pandemic metabolic syndromes will continue to worsen worldwide [4]. The health care costs associated with pandemic chronic metabolic syndromes may collapse the global health care system and must therefore be considered an urgent problem [4].

Over the past decade, 11β-hydroxysteroid dehydrogenase type 1 (11β-HSD1) has been reported to be one of the main factors responsible for metabolic syndromes [5]. Since 11β-HSD1 can amplify local glucocorticoids, it is correlated with the cause of obesity and insulin resistance behavior [6, 7]. Theoretically, 11β-HSD1 inhibitors can alleviate or even prevent some metabolic syndromes. Diabetes and hypertension are the common syndromes associated with metabolic diseases and are also frequently observed in ENDR-induced hyperadrenalism (commonly known as Cushing’s syndrome). Some encouraging and promising results have been found using 11β-HSD1 inhibitors as therapeutic agents, particularly for metabolic-related diseases [8–10]. Therefore, 11β-HSD1 inhibition appears to be a potential strategy to treat ENDR-induced hyperadrenalism.

In previous experiments [11], rats were supplied with NP-contaminated water during pregnancy (in utero) and lactation (neonatal) to mimic environmental contamination. The female pups showed elevated plasma corticosterone and aldosterone concentrations when they developed into adults. In addition, 11β-HSD1 activities and protein expression and aldosterone synthase activities were increased in the liver and adrenal tissues [11]. Since intracellular 11β-HSD1 regenerates active glucocorticoids (e.g., cortisol in humans and corticosterone in rodents) from inactive 11-keto forms in adipose, liver, and brain tissues, it will strengthen local cellular glucocorticoid action. Central obesity has been suggested to be due to amplified glucocorticoids in adipose tissue mediated by 11β-HSD1 [6, 12–14]. By using a transgenic mouse model, 11β-HSD1 overexpression resulted in visceral obesity, insulin-resistant diabetes, and hyperlipidemia [13]. However, the symptoms of insulin resistance, hyperglycemia, and dyslipidemia were not observed in 11β-HSD1-knockout mice [15].

These results seem to suggest that 11β-HSD1 inhibition is a potential therapeutic target for treating metabolic syndromes. In this study, we explore whether PF915275 (PF; 4′-cyano-biphenyl-4-sulfonic acid (6-amino-pyridin-2-yl)-amide), a selective inhibitor of 11β-HSD1 [16], can suppress ENDR-induced (i.e., NP-induced) hyperadrenalism and excess 11β-HSD1 activity. Since visceral obesity and hyperlipidemia are evident in 11β-HSD1-overexpressing mice [13], lipid metabolism is an essential aspect in studying 11β-HSD1 inhibition or activation. Researchers have shown that PPAR (peroxisome proliferator-activated receptor) ligands are highly associated with lipid metabolism. PPARs are a family of nuclear receptors, and specific PPAR ligands can activate certain types of PPARs. For example, PPAR type alpha (PPARα) ligand, i.e., fibrates, can activate transcription factors to enhance fatty acid oxidation. PPAR type gamma (PPARγ) ligand, i.e., thiazolidinediones, can reduce blood triglyceride and sugar levels and enhance lipogenesis via fatty acid synthesis. In this study, we explore the impacts of NP and PF on lipid metabolism through the PPAR system.

Demonstration of the therapeutic effectiveness of PF in a rat model (female adult rats exposed to NP during the developmental period to mimic environmental contamination received PF via oral gavage in this in vivo study) would offer hope for treating Cushing’s syndrome in humans. The first step was to verify whether NP increases adipogenesis in human adipose cells using in vitro methods. If an adipogenic effect is observed, then the 11β-HSD1 inhibitor requires verification by examining, for example, whether PF can alleviate or prevent this impact. Positive results from this study can serve as a basis for the development of clinical trials for endocrine-disrupting chemical (EDC)-induced metabolic syndrome.

## Methods

### Materials

NP was purchased from Fluka (Buchs, Switzerland) and dissolved in methanol to produce a 0.425 M stock solution. PF was purchased from Tocris Cookson Ltd. (Bristol, UK). The other chemicals were purchased from Sigma Chemical Co. (St. Louis, MO, USA). [^3^H]-Aldosterone and [^3^H]-corticosterone were purchased from Amersham Life Science Limited (Buckinghamshire, UK). Dr. D. M. Stocco (Department of Cell Biology and Biochemistry, Texas Tech University Health Sciences Center, Lubbock, TX, USA) generously provided the anti-steroidogenic acute regulatory (StAR) antibody. Anti-11β-hydroxysteroid dehydrogenase I (11β-HSD1), anti-fatty acid synthase (FASN), anti-proliferator-activated receptor α (PPARα), anti-proliferator-activated receptor γ (PPARγ), and anti-vinculin (Vinculin) antibodies were purchased from Abcam plc. (Cambridge, UK), GeneTex Inc. (San Antonio, TX, USA), Cayman Chemical (Ann Arbor, MI, USA), Cayman Chemical (Ann Arbor, MI, USA), and GeneTex Inc. (San Antonio, TX, USA), respectively. Anti-mouse and anti-rabbit IgG peroxidase-conjugated secondary antibodies were purchased from ICN Pharmaceuticals, Inc. (Aurora, OH, USA). Human preadipocytes were purchased from Cell Applications (San Diego, CA, USA). Preadipocyte medium (PAM) and preadipocyte differentiation medium (PADM) were purchased from ScienCell™ Research Laboratories (Carlsbad, CA, USA). A Lipid Oil Red O staining kit was purchased from BioVision Inc. (Milpitas, CA, USA).

### Animal treatment

#### Animals

Pregnant female Sprague-Dawley rats weighing 250–300 g were provided by the Animal Center of National Yang-Ming University. The pregnant female rats were divided into two groups, including NP mother (drinking NP-containing water (NP concentration 2 μg/ml) during the gestation and lactation periods) and vehicle control mother (drinking regular water). They were housed individually throughout their pregnancy until delivery, which occurred on days 20–22. The female pups of the NP group (mother drinking NP-containing water), the female pups of the vehicle control group (mother drinking regular water) and their mothers were housed in a temperature-controlled room (22 ± 1 °C) in the Animal Center of National Yang-Ming University with photoperiods of 14 h (light):10 h (dark) as previously described [17]. The lights were switched on at 6:30 a.m. and food and water were provided ad libitum. The number of female offspring from the same mother was 4–5 per cage. The female offspring of the NP group were randomly divided into an NP and an NP + PF group at 10 weeks of age. In the NP + PF group, the rats were given PF (4′-cyano-biphenyl-4-sulfonic acid (6-amino- pyridin-2-yl)-amide) at 0.36 mg/kg/ml (PF dissolved in DMSO solution) by oral gavage once per day, and the same amount of DMSO solution was administered orally to the vehicle and NP groups [17]. The treatment began at 10 weeks of age and lasted for 4 weeks. The adult rats (14 weeks old) were anesthetized with 100 mg/kg ketamine by intraperitoneal injection (ketamine dissolved in saline) and then sacrificed on the day after the last oral gavage. The sacrifice was implemented by the method of rapid decapitation. The procedures and precautions for sacrifice were the same as previously described [17]. In brief, the sacrifice began at 7:00 am to avoid adrenal rhythm variations, and sacrifice was sequentially performed in the vehicle group, NP group and NP + PF group. The process was repeated until all the rats were sacrificed. Blood and tissue collection from one rat required 10–15 min, and approximately 3–3.5 h were required to complete sample collection. Trunk blood was collected and plasma samples were separated and stored at − 20 °C until analysis. The liver, adipose tissue, and adrenal glands were immediately dissected and stored at − 80 °C. The rat carcasses were handled by the Animal Center of National Yang-Ming University.

All animal protocols used in this study were approved by the Institutional Animal Care and Use Committee of the National Yang-Ming University. All animals received humane care in compliance with the Principles of Laboratory Animal Care and the Guide for the Care and Use of Laboratory Animals, published by the National Science Council, Taiwan, R.O.C.

#### Enzyme activity assays

The oxidoreductase activity assay for 11β-HSD1 was performed by measuring corticosterone produced from 11-dehydrocorticosterne [17–19]. Specific activities were expressed as nanograms of corticosterone formed per hour per gram of protein.

The 11β-hydroxylase activity of the adrenal cortex was measured from the corticosterone produced from deoxycorticosterone treatment [20]. Specific activities were expressed as nanograms of corticosterone formed per hour per microgram of adrenal cortex protein, while the aldosterone measurements after corticosterone treatment [20] represented the aldosterone synthase activity of the adrenal capsule, which was expressed as the amount of aldosterone (usually in nanograms) formed per hour per microgram of adrenal capsule protein.

#### Corticosterone and aldosterone RIA

Corticosterone or aldosterone in plasma was extracted using diethyl ether as previously described [20]. After the diethyl ether evaporated, assay buffer was added to dissolve the corticosterone or aldosterone. RIA can be used to determine both the concentration of corticosterone [21] and the concentration of aldosterone [22] in the plasma or in media. The plasma corticosterone levels were measured by RIA using antiserum (PSW#4–9), the sensitivity of the corticosterone RIA was 5 pg/tube, and the intra- and inter-assay coefficients of variation were 3.3% (*n* = 5) and 9.2% (*n* = 4), respectively. Additionally, an RIA using JJC-088 antiserum was used to measure plasma aldosterone levels. The sensitivity of the aldosterone RIA was 4 pg/tube, and the intra- and inter-assay coefficients of variation were 3.9% (*n* = 5) and 8.2% (*n* = 4), respectively.

### Cell culture and treatment

#### Cell culture

Human preadipocytes were purchased from Cell Applications (San Diego, CA, USA), maintained in preadipocyte medium (PAM, ScienCell™ Research Laboratories, Carlsbad, CA, USA) and incubated at 37 °C in 5% CO_2_. For differentiation, human preadipocytes were seeded into plates or dishes (48,000 cells/cm^2^ in PAM) for 2 days, and then the preadipocytes were incubated with preadipocyte differentiation medium (PADM, ScienCell™ Research Laboratories, Carlsbad, CA, USA) for 6 days by changing the medium every 3 days in the presence or absence of NP or PF at different concentrations. After differentiation, the cells were harvested for the tests described below.

#### Preadipocyte proliferation

Human preadipocytes were seeded at 4.5 × 10^3^ cells/well in a 96-well plate. The cells were incubated in PAM in the presence of NP or PF for 24 and 72 h. After incubation, the cells were treated with 20 μl of MTT (dissolved in PBS (pH 7.4)) assay reagents (5 mg/ml) for 4 h, and the resulting purple formazan crystals were solubilized in 150 μl of DMSO. The absorbance was measured at 570 nm on a Sunrise™ microplate reader (Tecan Trading AG, Switzerland). The percentages of viable cells were calculated in the NP- or PF-treated groups relative to the control cells (cells incubated with PAM for 24 h).

#### Cell viability

To assess cell viability after NP or PF treatment, human preadipocytes were seeded onto 96-well plates at a density of 4.5 × 10^3^ cells/well and differentiated with PADM in the presence of NP or PF for 6 days. After incubation, cell viability was quantitatively assessed using the MTT assay. The percentages of viable cells were calculated in the NP- or PF-treated groups relative to that in the control group (cells differentiated with PADM alone for 6 days).

#### Oil red O staining

To assess lipid accumulation with NP or PF treatment, human preadipocytes were seeded onto 24-well plates at a density of 4.5 × 10^4^ cells/well and differentiated with PADM in the presence of NP or PF for 6 days. The formation of lipid droplets in adipocytes was observed with a Lipid Oil Red O staining kit (BioVision Inc., Milpitas, CA, USA) according to the manufacturer’s protocol. To measure lipid accumulation in adipocytes, the cells were washed with 60% isopropanol 3 times, and the completely dried cells were shaken with 100% isopropanol for 5 min. The dissolved lipid was quantified by measuring the absorbance at 492 nm. The results were expressed as the lipid accumulation relative to that in the control cells (cells differentiated with PADM only for 6 days).

#### Tissue and cell protein extraction

Frozen liver, adrenal, and adipose tissue samples or human preadipocytes were lysed in homogenizing buffer (containing 65 mM Tris-base, 154 mM sodium chloride, 1% NP-40, 6 mM sodium deoxycholate, 1 mM ethylenediaminetetraacetic acid (EDTA), 1 mM phenylmethylsulfonyl fluoride (PMSF), 5 mg/l aprotinin, 5 mg/l leupeptin, 5 mg/l pepstatin, 1 mM sodium orthovanadate, 1 mM sodium fluoride, and 1% proteinase inhibitor cocktail (pH 7.4) for the liver, adipose tissues and human preadipocytes, and containing 1.5% N-lauroylsarcosine sodium, 2.5 mM Tris-base, 1 mM EDTA, 0.68% PMSF, and 2% proteinase inhibitor cocktail (pH 7.8) for the adrenal tissue, the adrenal capsule, and the adrenal cortex) for 30 min on ice. The obtained homogenates were centrifuged at 13,000 × *g* for 30 min at 4 °C, and the supernatants were sampled. The protein concentration was evaluated using the Bradford assay [23] with bovine serum albumin (BSA) as the standard.

#### Western blot analysis

The levels of 11β-HSD1 (for liver and adipose tissues), PPARα (for liver, adipose tissues, adrenal tissue, and human preadipocytes), PPARγ (for adipose tissue and human preadipocytes), FASN (for human preadipocytes) and StAR (for adrenal tissue) protein expressions were determined by western blot analysis [24]. For western blotting, samples containing equal amounts of protein (liver protein (100 μg), adipose protein (200 μg), adrenal protein (100 μg), or human preadipocyte protein (50 μg)) were loaded on a 6 or 10% acrylamide sodium dodecyl sulfate-polyacrylamide gel for electrophoresis (SDS-PAGE). After electrophoresis, the relevant proteins were detected on blots by specific antibodies, and signals were developed using enhanced chemiluminescence (ECL western blotting detection reagents, Amersham Pharmacia Biotech, Buckinghamshire, UK) and the Luminescent Image Analyzer Las-4000 (Fuji-Film, Stamford, CT, USA). Additionally, the MultiGauge program (Fuji-Film) was used to quantify proteins, and the protein signals were normalized against the glyceraldehyde-3-phosphate dehydrogenase (GAPDH) or vinculin protein signal.

#### RNA extraction and quantitative real-time PCR

Total RNA from the liver and human preadipocytes was extracted using TRIzol reagent (Molecular Research Center, Inc., Cincinnati, OH, USA). The concentration of RNA was measured by the OD260, and the purity of RNA was evaluated by measuring the ratio of A260/A280 (selecting values above 1.8) with the aid of a Nanodrop (Thermo Scientific, Waltham, MA, USA). cDNA was synthesized with a cDNA synthesis kit (Bioman Scientific Co., Ltd., Taipei, Taiwan). Real-time PCR reactions were performed with a SYBR Green PCR Master Mix kit (Applied Biosystems, Inc., Foster City, CA, USA) and 50 ng of cDNA in an ABI real-time PCR detection system. Relative mRNA expression was determined by the 2-△△Ct method normalized to *GAPDH* or *18S rRNA*, which were used as housekeeping genes. Additionally, the vehicle values were normalized to a group average of 1.0. PCR product purities were verified by melting curve analysis. The primers were synthesized by Tri-I Biotech Inc. (Taipei, Taiwan) (the sequences for the primers are shown in Table 1).

**Table 1:** Real-time PCR primers

Gene	Sequence (5′ to 3′ forward)	Sequence (5′ to 3′ reverse)
*11*β*-HSD1*	CCTCCCCGTCCTGGTGCTCT	TGCTCCGAGTTCAAGGCAGCG
*GAPDH*	TGTGAACGGATTTGGCCGTA;	GATGGTGATGGGTTTCCCGT
*PPAR*α	GCTCTGAACATTGGCGTTCG	TCAGTCTTGGCTCGCCTCTA
(h) *FASN*	CGCGT GGCCGGCTACTCCTAC	CGGCTGCCACACGCTCCTCT
(h) *PPAR*α	CAGAACAAGGAGGCGGAGGTC	TTCAGGTCCAAGTTTGCGAAGC
(h) *PPAR*γ	AGGCGAGGGCGATCTTGACAG	GATGCGGATGGCCACCTCTTT
(h) *18 s rRNA*	TGCCATGTCTAAGTACGCACG	TTGATAGGGCAGACGTTCGA

#### Statistical analysis

The results are expressed as the mean ± S.E.M. Comparisons across a variety of treatments were performed using one-way ANOVA followed by post hoc analysis of Fisher’s least significant difference (LSD) or Dunnett’s post hoc analysis. Values of *p* < 0.05 were considered statistically significant.

## Results

### PF suppresses NP-induced hyperadrenalism and adipogenesis

#### Plasma levels of steroid hormones

**Fig. 1:**
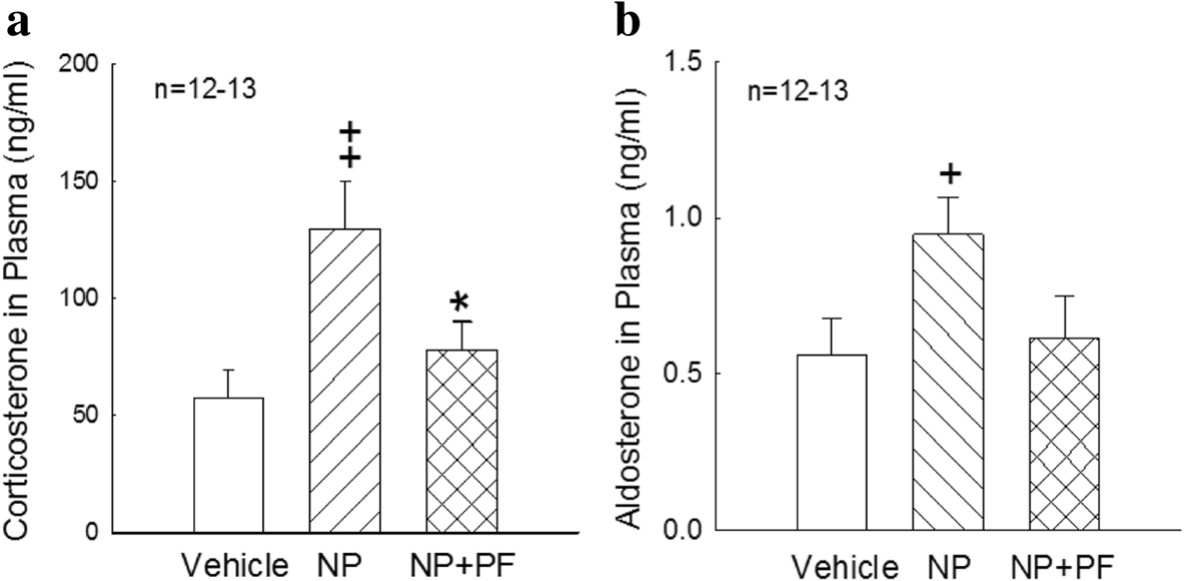
Corticosterone (**a**) and aldosterone (**b**) concentrations in plasma from vehicle-, NP- and NP + PF-treated female rats (Veh *n* = 12, NP *n* = 12, NP + PF *n* = 13). These concentrations were measured by RIA. The values are presented as the means ± S.E.M. +, ++: *p* < 0.05, 0.01, NP group compared with the vehicle group. *: *p* < 0.05, NP + PF group compared with the NP group

Developmental exposure to NP significantly increased plasma corticosterone concentrations in female offspring (*p* < 0.01, Fig. 1a), and PF significantly decreased the corticosterone concentrations in the NP + PF group compared with those in the NP group (*p* < 0.05, Fig. 1a).

The plasma aldosterone concentrations also significantly increased in the NP group (*p* < 0.05, Fig. 1b) compared with those in the vehicle group. PF appeared to reduce the plasma aldosterone concentrations, although not significantly, compared with those in the NP group (*p* < 0.1, Fig. 1b).

#### Liver

**Fig. 2:**
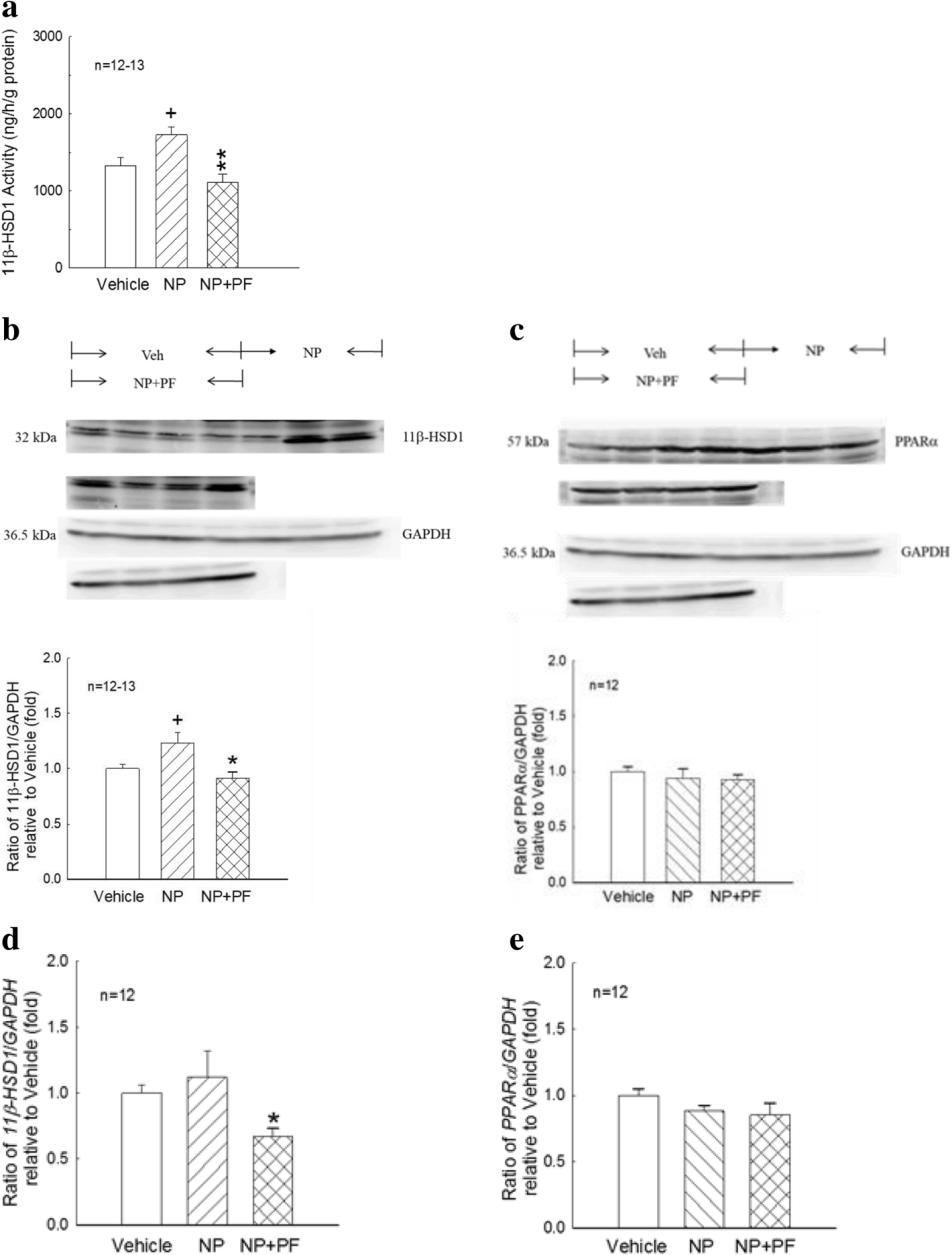
11β-HSD1 and PPARα activity and expression in the liver from vehicle-, NP- and NP + PF-treated female rats. **a**: The 11β-HSD1 reductase activity was measured in the liver from vehicle-, NP- and NP + PF-treated female rats using 11-dehydrocorticosterone as a substrate in the presence NADPH. Values are shown as the means ± S.E.M. **b** and **c**: Expression of 11β-HSD1 (32 kDa) and PPARα (57 kDa) in livers from vehicle-, NP- and NP + PF-treated female rats were determined by western blot analysis. The representative immunoblot was shown in the upper part, and densitometry for 11β-HSD1 or PPARα normalized to GAPDH was shown in the bottom part. The values are presented as the means ± S.E.M. and are presented as the fold-change compared to the vehicle group. **d** and **e**: The mRNA levels of *11β-HSD1* and *PPARα* in the liver were determined by real-time PCR. The values are presented as the means ± S.E.M. and are presented as the fold-change relative to the vehicle group. +: *p* < 0.05, NP group compared with vehicle group. *, **: *p* < 0.05, 0.01, NP + PF group compared with the NP group

Developmental exposure to NP significantly increased 11β-HSD1 activity in the liver (*p* < 0.05, Fig. 2a), and treatment with PF significantly decreased 11β-HSD1 activity in the liver (*p* < 0.01, Fig. 2a) compared with that in the NP group. The 11β-HSD1 and PPARα protein levels in the liver were determined by western blot analysis (Fig. 2b, c). The 11β-HSD1-to-GAPDH protein ratio was significantly increased in the NP group (*p* < 0.05, Fig. 2b) compared with that in the vehicle group but significantly decreased in the NP + PF group (*p* < 0.05, Fig. 2b) compared to that the NP group. The PPARα-to-GAPDH protein ratio was not significantly different among the vehicle, NP, and NP + PF groups (Fig. 2c). The *11β-HSD1* and *PPARα* mRNA levels in the liver were determined by real-time PCR (Fig. 2d, e). NP exposure during the developmental period did not significantly affect the mRNA expression of *11β-HSD1* and *PPARα* in the liver compared to that in the vehicle group (Fig. 2d, e). Oral administration of PF in the NP + PF group significantly decreased the mRNA expression of *11β-HSD1* (*p* < 0.05, Fig. 2d) in the liver compared with that in the NP group.

#### Adipose tissue

**Fig. 3:**
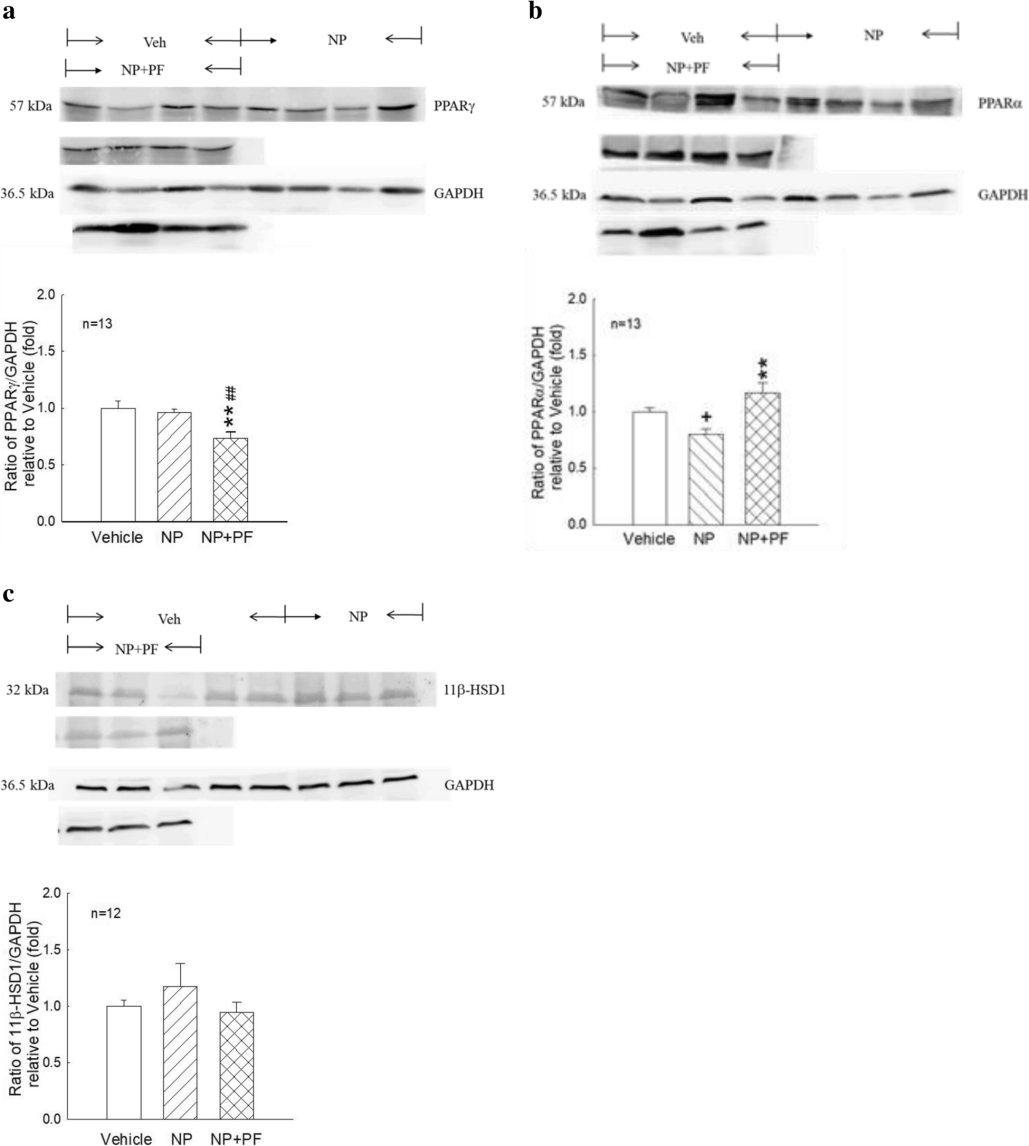
PPARγ, PPARα and 11β-HSD1 protein expression in the adipose tissues from vehicle-, NP- and NP + PF-treated female rats. **a**, **b** and **c**: PPARγ(57 kDa), PPARα (57 kDa) and 11β-HSD1 (32 kDa) protein levels in vehicle-, NP- and NP + PF-treated female rats were determined by western blot analysis. The representative immunoblot was shown in the upper part, and densitometry for PPARγ, PPARα or 11β-HSD1 normalized to GAPDH was shown in the bottom part. The values are presented as the means ± S.E.M. and are presented as the fold-change relative to the vehicle group. +: *p* < 0.05, NP group compared with the vehicle group. **: *p* < 0.01, NP + PF group compared with the NP group. ##: *p* < 0.01 NP + PF group compared with the vehicle group

NP exposure decreased PPARα protein expression (*p* < 0.05, Fig. 3b) but did not affect PPARγ protein expression (Fig. 3a). PF treatment in the NP + PF group significantly increased PPARα protein expression (*p* < 0.01, Fig. 3b) but decreased PPARγ protein expression (*p* < 0.01, Fig. 3a) compared with that in either the vehicle or NP group. The 11β-HSD1-to-GAPDH protein ratio was not significantly different among the vehicle, NP, and NP + PF groups (Fig. 3c).

#### Adrenal glands

**Fig. 4:**
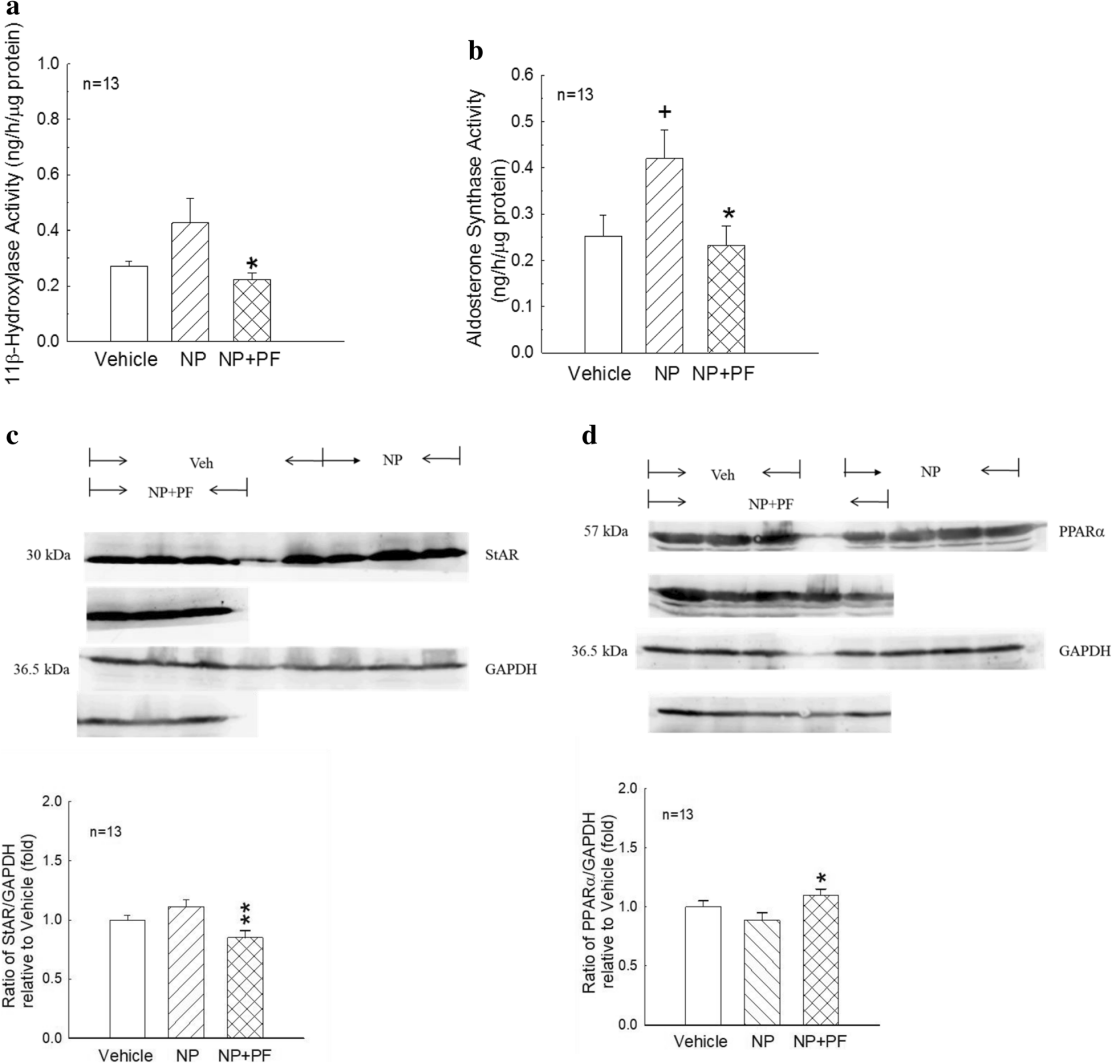
The adrenals from vehicle-, NP-, and NP + PF-treated female rats. **a**: The 11β-hydroxylase activity was measured in the adrenal cortex using deoxycorticosterone as a substrate. **b**: The aldosterone synthase activity was measured in the adrenal capsule using corticosterone as a substrate. Values are shown as the means ± S.E.M. **c** and **d**: Expression levels of StAR (30 kDa) and PPARα (57 kDa) in the adrenals from vehicle-, NP- and NP + PF-treated female rats were determined by western blot analysis. The representative immunoblot was shown in the upper part, and densitometry for StAR or PPARα normalized to GAPDH was shown in the bottom part. The values are presented as the means ± S.E.M. and are presented as the fold-change relative to the vehicle group. +: *p* < 0.05, NP group compared with the vehicle group. *, **: *p* < 0.05, 0.01, NP + PF group compared with the NP group

NP exposure significantly increased aldosterone synthase activity in the adrenal capsule (*p* < 0.05, Fig. 4b). In the NP + PF group, PF significantly decreased 11β-hydroxylase activity in the adrenal cortex (*p* < 0.05, Fig. 4a) and decreased aldosterone synthase activity in the adrenal capsule (*p* < 0.05, Fig. 4b) compared with the activity levels in the NP group. The levels of StAR and PPARα proteins in the adrenal tissues were determined by western blot analysis (Fig. 4c, d). NP exposure apparently increased StAR protein expression, although not significantly, (Fig. 4c), whereas PF significantly decreased StAR protein expression (*p* < 0.01, Fig. 4c) compared with the expression levels in the NP group. NP exposure appeared to decrease PPARα protein expression, although not significantly (Fig. 4d), compared with that in the vehicle group, while PF significantly increased PPARα protein expression (*p* < 0.05, Fig. 4d) compared with that in the NP group.

### Human preadipocyte culture

#### Effects of NP and PF on cell proliferation, cell viability and lipid accumulation

**Fig. 5:**
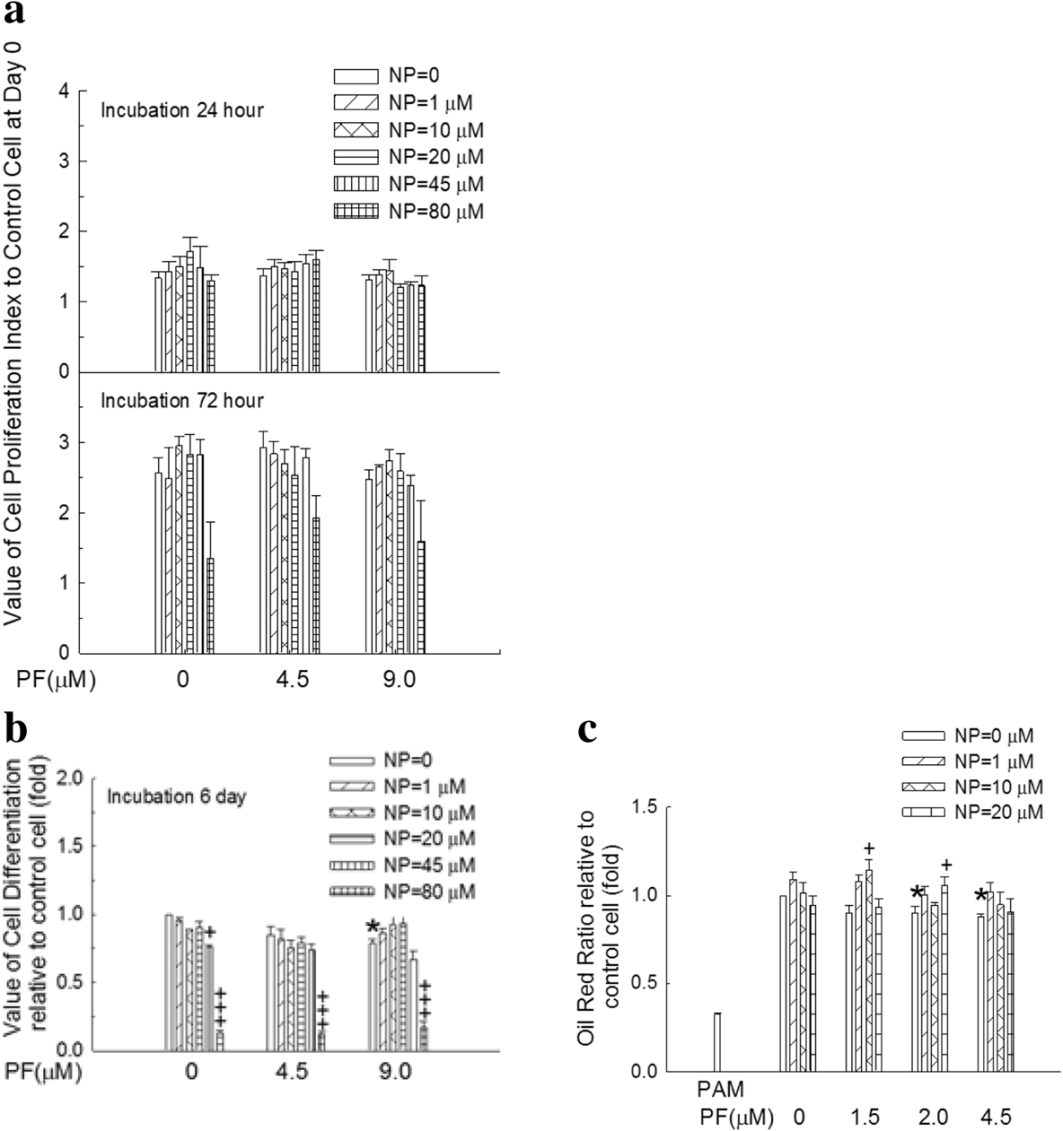
Effects of NP and PF on cell proliferation, cell viability and lipid accumulation. **a**: Human preadipocytes were seeded onto 96-well plates at a density of 4.5 × 10^3^ cells/well and treated with NP combined with PF for 24 or 72 h. MTT reagent was added to the medium. After 4 h of incubation, the medium was aspirated, and 150 μl DMSO was added to each well. The absorbance was read at 570 nm. **b**: Human preadipocytes were incubated in differentiation medium (PADM) in the presence of NP combined with PF. After 6 days, MTT assay was performed to assess the cell viability. c: Human preadipocytes were incubated in differentiation medium (PADM) in the presence of NP combined with PF for 6 days. Oil Red O staining was performed, and stained lipids were extracted and quantified by measuring absorbance at 492 nm. Values are means of 3 independent experiments, are shown as the mean ± S.E.M, and are presented as the fold-change relative to control cells. a: cells incubated with PAM for 24 h; **b** and **c**: cells differentiated with only PADM for 6 days. +, +++: *p* < 0.05, 0.005, NP effect with the same concentration of PF. *: *p* < 0.05, PF effect with the same concentration of NP

Human preadipocytes were grown in PAM supplemented with different concentrations of NP combined with PF for 24 and 72 h. As shown in Fig. 5a, the MTT assay revealed that neither NP nor PF affected preadipocyte proliferation. Human preadipocytes were induced to differentiate in the presence of NP (0–80 μM) combined with PF (0–9 μM) for 6 days. The MTT assay revealed that NP at concentrations of 1–20 μM and PF at concentrations of 4.5 μM did not affect cell viability (Fig. 5b). Therefore, these concentration ranges of NP or PF were selected for further experiments. Human preadipocytes underwent morphological changes from spindle-like features to a round shape and accumulated intracellular lipids after differentiation induction. To examine the accumulation of intracellular lipids, preadipocytes were cultured in PADM for 6 days in the presence of NP (0–20 μM) combined with PF (0–4.5 μM) (Fig. 5c). Lipid accumulation was measured by Oil red O staining. PF at 2 or 4.5 μM decreased lipid accumulation (*p* < 0.05, Fig. 5c). NP at 10 μM increased PF-inhibited lipid accumulation at 1.5 μM PF (*p* < 0.05, Fig. 5c). NP at 20 μM increased PF-inhibited lipid accumulation at 2 μM PF (*p* < 0.05, Fig. 5c). NP alone did not increase lipid accumulation (Fig. 5c), but it increased PF-inhibited lipid accumulation.

#### Effect of human preadipocyte exposure to NP before differentiation

To elucidate any epigenetic modifications or “memory” effects, human preadipocytes were exposed to NP before differentiation (P exposure). Preadipocytes were seeded into dishes (3.5 × 10^5^ cells in PAM) for 36 h, and then the medium was changed to PAM in the presence of 20 μM NP for 12 h. After 12 h, preadipocytes were cultured in PADM (without NP) for 6 days in the presence of PF (0, 2 or 4.5 μM). The PPAR pathway and FASN was examined at the mRNA and protein levels.

**Fig. 6:**
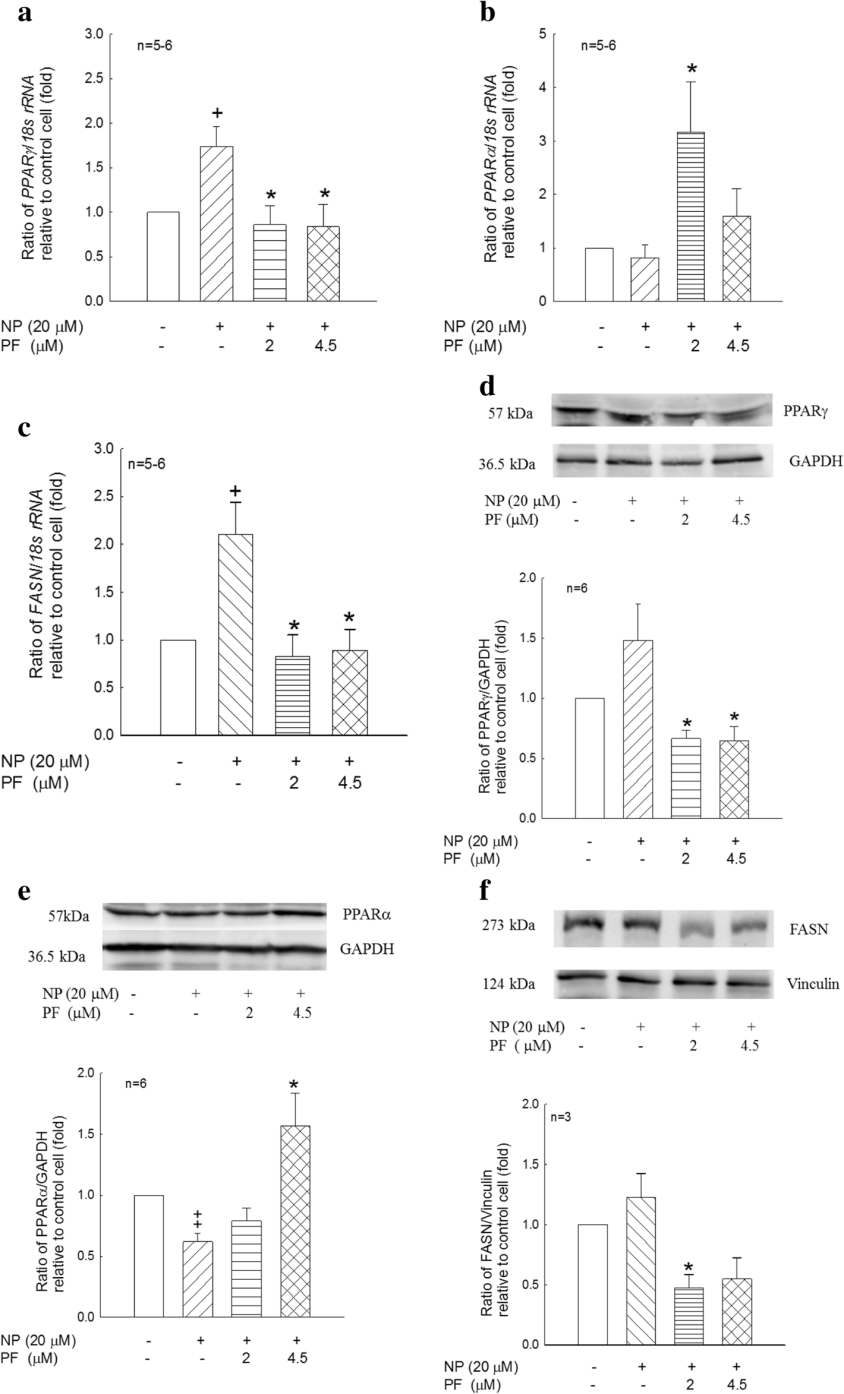
Effects of human preadipocyte exposure to NP before differentiation (P exposure) on mRNA and protein expression levels by real-time PCR and western blot analysis. Human preadipocytes were incubated in PAM in the presence of NP (0 or 20 μM) for 12 h. After exposure to NP, the medium was changed to differentiation medium (PADM) in the presence of PF915275 (0, 2 or 4.5 μM). **a**, **b** and **c**: After 6 days of incubation, *PPARγ*, *PPARα* and *FASN* mRNA levels in the cells were determined by real-time PCR analysis, and the values were normalized against *18 s rRNA*. **d**, **e**, **f**: After 6 days of incubation, PPARγ (57 kDa), PPARα (57 kDa) and FASN (273 kDa) protein levels in the cells were determined by western blot analysis. The representative immunoblot was shown in the upper part, and densitometry for PPARγ, PPARα or FASN normalized to GAPDH (for PPARγ, PPARα) or Vinculin (for FASN) was shown in the bottom part. The values, which are means of independent cultures, are shown as the means ± S.E.M. and are presented as the fold-change relative to control cells (cells were not primed with NP and differentiated with PADM only). +, ++: *p* < 0.05, 0.01, cells with priming with NP and differentiation with PADM compared with control cells. *: *p* < 0.05, cells with priming with NP and differentiation with PADM containing PF compared with cells with priming with NP and differentiation with PADM only

Priming with NP significantly increased the *PPARγ* and *FASN* (*p* < 0.05, Fig. 6a, c) mRNA expression levels, whereas PF treatment significantly decreased their mRNA expression levels (*p* < 0.05, Fig. 6a, c). In contrast, PF treatment significantly increased *PPARα* mRNA expression (*p* < 0.05, Fig. 6b), and priming with NP significantly decreased PPARα protein expression (*p* < 0.01, Fig. 6e), but PF significantly reversed this phenomenon (*p* < 0.05, Fig. 6e). NP priming enhanced the protein expression of PPARγ and FASN, but not significantly. With the addition of PF, PPARγ and FASN protein expression levels were significantly reduced (*p* < 0.05, Fig. 6d, f). These data suggested that human preadipocytes exposed to NP before differentiation exhibited effects after differentiation. The differentiated adipocytes exhibited memory of exposure to NP and showed increased adipogenesis and decreased lipolysis. Treatment with PF alleviated these effects at the differentiated adipocyte level.

#### Effects of NP and/or PF on protein and RNA expression after induced adipocyte differentiation

To elucidate the molecular mechanisms by which NP and PF affected adipogenesis after induction into adipocytes, human preadipocytes were seeded into dishes (3.5 × 10^5^ cells in PAM) for 48 h, and the medium was changed to PADM for 6 days in the presence of NP (0 or 20 μM) combined with PF (0, 2 or 4.5 μM). After the induction period, the preadipocytes differentiated into adipocytes. Real-time PCR and western blotting analyses were performed to examine the effects of PF and NP on the expression of adipogenesis-related mRNA and proteins.

**Fig. 7:**
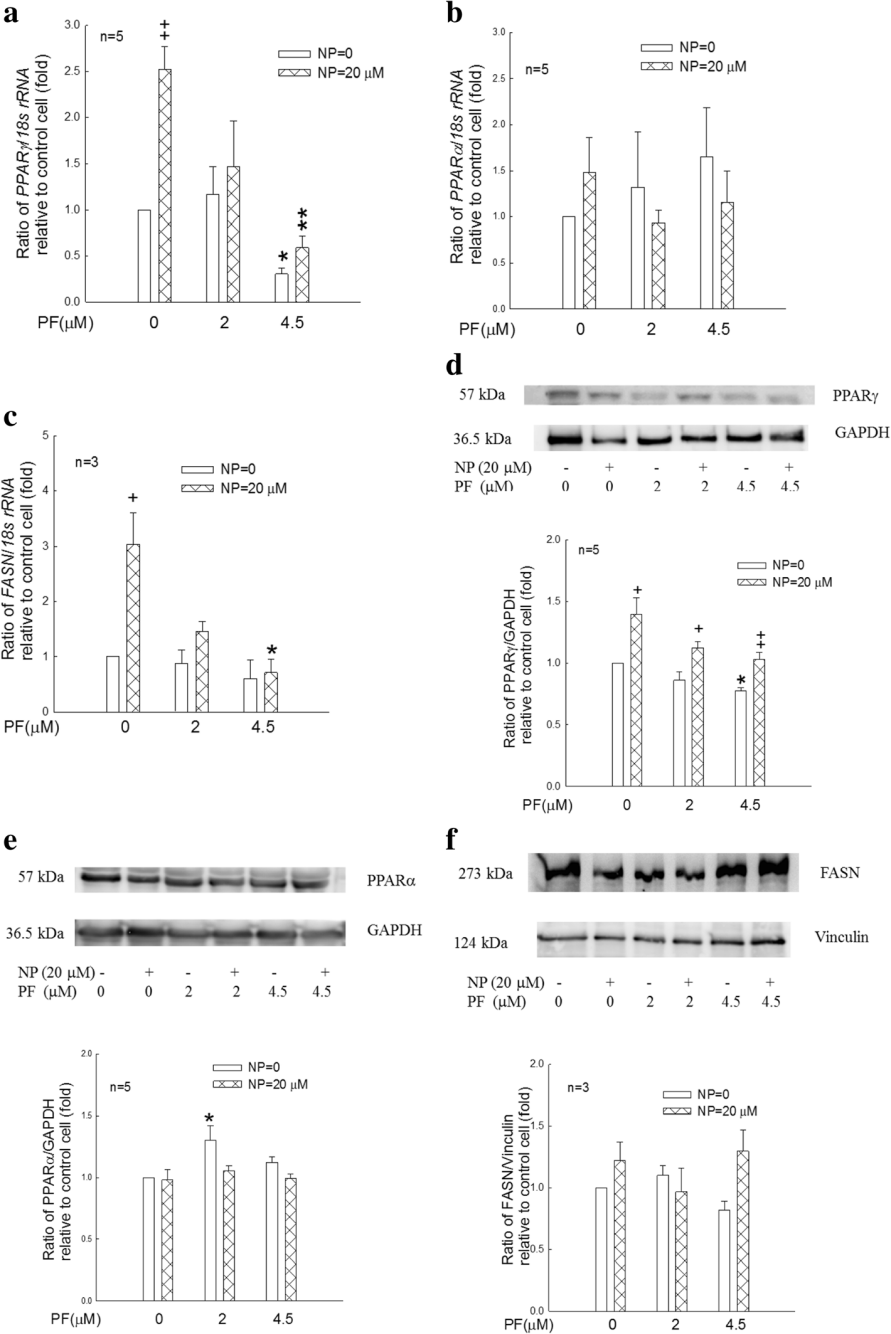
Effects of NP and PF on mRNA and protein expression levels by real-time PCR and western blot analysis. Human preadipocytes were incubated in differentiation medium (PADM) in the presence of NP (0 or 20 μM) combined with PF915275 (0, 2 or 4.5 μM). **a**, **b** and **c**: After 6 days of incubation, *PPARγ*, *PPARα* and *FASN* mRNA levels in the cells were determined by real-time PCR analysis, and the values were normalized against *18 s rRNA*. **d**, **e** and **f**: After 6 days of incubation, PPARγ (57 kDa), PPARα (57 kDa) and FASN (273 kDa) protein levels in the cells were determined by western blot analysis. The representative immunoblot was shown in the upper part, and densitometry for PPARγ, PPARα or FASN normalized to GAPDH (for PPARγ, PPARα) or Vinculin (for FASN) was shown in the bottom part. The values, which are means of independent cultures, are presented as the means ± S.E.M. and represent the fold-change relative to control cells (cells differentiated with PADM only). +, ++: *p* < 0.05, 0.01, NP effect with the same concentration of PF. *, **: *p* < 0.05, 0.01, PF effect with the same concentration of NP

For mRNA expression, adipocytes treated with 20 μM NP had significantly increased *PPARγ* (*p* < 0.01, Fig. 7a) and *FASN* mRNA expression levels (*p* < 0.05, Fig. 7c). Treatment with 4.5 μM PF significantly decreased basal (*p* < 0.05) and NP-induced *PPARγ* mRNA expression (*p* < 0.01, Fig. 7a) and decreased NP-induced *FASN* mRNA expression (*p* < 0.05, Fig. 7c). Adipocytes treated with 20 μM NP had increased PPARγ protein expression (*p* < 0.05, Fig. 7d), while PF reduced PPARγ protein expression (*p* < 0.05, Fig. 7d). PF increased PPARα protein expression, but NP had no significant effect (*p* < 0.05, Fig. 7e). Based on the human preadipocyte model, these data suggested that NP increased lipogenesis via PPARγ and FASN mRNA/protein expression whereas PF counteracted the NP effects.

## Discussion

In this study, water containing NP at a 2 μg/ml concentration mimicked environmentally relevant exposure conditions. Water consumption among female rats during pregnancy and late lactation ranges from approximately 25 ml/day to 100 ml/day. For calculation purposes, NP water consumption was assumed to be 50 ml/day during pregnancy and 100 ml/day during late lactation. Therefore, the highest daily NP exposure level was estimated to be approximately 400 μg/kg/day during pregnancy and 800 μg/kg/day during late lactation. The examined dose is between the tolerable intake level reported by the Danish Institute of Safety & Toxicity (i.e., 5 μg/kg/day) [25] and the previously reported no-adverse-effects-observed level (i.e., 45–50 mg/kg/day) [26]. The exposure dose used is the lowest amount relative to other dietary exposure levels [27–30]. Courtney et al. [16] proposed that healthy humans could safely tolerate an oral dose of 0.3 mg/kg/day of PF for 2 weeks and concluded PF was a safe and selective 11β-HSD1 inhibitor. Bhat et al. [31] reported that oral administration of 3 mg/kg of PF could inhibit 11β-HSD1 activity in Cynomolgus monkeys. In this in vivo study, the oral dose of PF was higher and the treatment period was longer than those in the studies of Bhat et al. [31] and Courtney et al. [16] for two reasons: 1) the metabolism of rats is higher than that of humans and monkeys, and 2) the two studies from Courtney et al. [16] and Bhat et al. [31] examined the safety and effects of PF in healthy subjects, whereas we examined the effect of PF in affected animals. This study sought to define a therapeutic treatment for rats with induced hyperadrenalism. Other 11β-HSD1 inhibitors are under investigation in therapeutic studies. Wang et al. [32] reported that treating diet-induced obese mice with oral BVT 2733 (a selective 11β-HSD1 inhibitor) at 100 mg/kg/day for 1 week could attenuate obesity and inflammation. Hu et al. [33] also proposed that the lipid profiles of high-fat diet-treated rats could be improved by oral gavage with curcumin (a selective 11β-HSD1 inhibitor) at 200 mg/kg/day for 2 months. As shown in Fig. 5b, a concentration of NP or PF higher than 20 μM or 4.5 μM, respectively, could induce cytotoxicity in human preadipocytes. Therefore, the concentrations of NP and PF used were not greater than 20 μM and 4.5 μM, respectively, in this in vitro study.

Nonylphenol ethoxylate is a non-ionic surfactant with many applications in industrial, agricultural and household products. NP is the degradation product of nonylphenol ethoxylate and an environmental pollutant that drastically disrupts the endocrine system [34]. Most studies regarding its endocrine-disrupting effects have mainly focused on its estrogenic activity [35]. However, our study shows that NP can induce hyperadrenalism both in vitro and in vivo [20, 36, 37]. In addition to the adrenal axis effect, we also observed that local tissues, i.e., adipose tissue, can convert the non-active 11-keto form to an active corticoid through 11β-HSD1. This action does not appear to be under hypothalamo-hypophyseal-adrenal regulation [20]. Increasing cellular 11β-HSD1 activity is associated with obesity and metabolic syndrome [5, 38, 39], which has been identified as an intracellular Cushing’s state [40].

Hyperadrenalism was observed in female offspring based on elevated plasma corticosterone levels and decreased sensitivity of the hypothalamic-pituitary-adrenal (HPA) negative feedback system [20]. These observations corresponded to a phenomenon of HPA axis activation by prenatal stress or glucocorticoid exposure during pregnancy [39, 41]. We also previously observed that exposure of female pups to NP during the developmental period not only elevated blood corticoid concentrations but also increased 11β-HSD1 protein expression and enzymatic activity in the liver [11]. These findings are consistent with the results in this study. Epigenetic modifications during the sensitive developmental period can induce “fetal origins of adult disease”. These epigenetic modifications by DNA methyltransferases can be inherited [42]. NP-induced hyperadrenalism, if left untreated, can impact an individual’s health for life. We previously reported [11] that two generations are required for female rats (the F_3_ generation recovers compared to the NP-affected F_1_ generation) to recover from NP-induced hyperadrenalism. The logical treatment plan for the NP-affected F_1_ generation consists of counteracting the increased 11β-HSD1 activity due to its irreversible effects on epigenetic modifications. One potential agent is a selective inhibitor of 11β-HSD1 called PF, which is currently undergoing the initial phase I clinical trial test. Thus far, PF has been shown to be a safe and effective treatment for metabolic syndrome [16, 31, 43]. In this study, oral gavage with PF significantly decreased corticosterone release in plasma (Fig. 1a) and decreased 11β-HSD1 enzyme activity and protein and mRNA expression in the liver (Fig. 2a, b and d). StAR protein can transfer cholesterol from the outer mitochondrial membrane to the inner mitochondrial membrane. Clark et al. [44] purified, cloned and sequenced the StAR protein. In our previous in vivo [11, 20] and in vitro [36] studies, NP did not increase StAR protein expression in the adrenal gland. These findings are consistent with the results in this study (Fig. 4c). Additionally, PF drastically decreased 11β-hydroxylase activity, aldosterone synthase activity and StAR protein expression in the adrenal glands of female rat offspring with developmental exposure to NP (Fig. 4a-c). These results show that PF can alleviate or reduce NP-induced hyperadrenalism in rats.

In vivo, chronic glucocorticoid exposure stimulates both adipogenesis and lipolysis in visceral adipose tissue; adipogenesis increases adipose tissue mass and lipolysis increases circulating free fatty acids and ectopic lipid storage in the liver and skeletal muscle [45]. Both in vivo and in vitro studies have shown that glucocorticoids stimulate lipolysis via hormone-sensitive lipase [46, 47] and that the resultant free fatty acids may contribute to insulin resistance. Glucocorticoids acting with insulin can regulate lipid homeostasis in human preadipocyte cells [48]. Peroxisome proliferator-activated receptor γ (PPARγ), which is primarily expressed in adipose tissues engaging in adipogenesis, plays an important role in adipocyte differentiation, inflammation, glucose homeostasis and insulin signaling [49]. Octylphenol is another alkylphenol similar to NP. Treatment of C3H10T1/2 cells with octylphenol induces PPARγ expression to inhibit osteoblast differentiation, causing a lineage shift towards adipocytes [50, 51]. Hao et al. [52] showed that NP induced *PPARγ* expression in mouse 3 T3-L1 preadipocytes, and NP increased *PPARγ* expression in mice injected with NP after 24 h. In this study, no significant differences in PPARγ protein expression levels were found between the vehicle and NP groups (Fig. 3a). However, PF treatment significantly decreased the protein expression of PPARγ in adipose tissue (Fig. 3a). In human preadipocytes, P exposure of NP significantly increased *PPARγ* mRNA expression, and PF decreased NP-induced increases in PPARγ mRNA and protein expression to inhibit adipogenesis. These observations may suggest that the effects of NP on human (Fig. 6a) and rodent tissues [52] may be mediated through similar mechanisms at the cellular level. In this in vitro study, P exposure was designed to serve as a model for the human neonatal (not fully differentiated) period. The results showed that preadipocytes had “memory” of NP exposure during the pre-differentiation period, possibly suggesting that P exposure triggered epigenetic modifications or long-term consequences of molecular or biochemical events. The results obtained from human preadipocytes corresponded to the rat model of developmental exposure to NP, which resulted in lifelong hyperadrenalism. This observation supports Barker’s hypothesis of the “fetal origins of adult disease” [53]. C exposure was designed as a model to examine the effects of NP exposure during the human adult period. C exposure of NP in human preadipocytes significantly increased PPARγ mRNA and protein expression levels, and PF decreased NP-induced PPARγ mRNA and protein expression (Fig. 7a, d). NP exposure appeared to trigger hyperadrenalism [20]. Whether hyperadrenalism induced by NP exposure during the adult stage had “memory” effects was unclear. Nevertheless, both life stages (P exposure and C exposure) showed adipogenic consequences of exposure to NP. These observations support the Baillie-Hamilton theory [1] that environmental ENDRs are partially responsible for pandemic metabolic syndromes. The effects of the 11β-HSD1 inhibitor PF reflect the possibility of treating and alleviating the impacts of environmental pollution.

Fatty acid synthase (FASN) is a key enzyme in lipogenesis. Sprague-Dawley rat pups fed with a high-carbohydrate diet exhibited obesity later in life and overexpressed FASN in the liver and adipose tissue [54]. NP exposure by either the P or C protocol can increase *FASN* mRNA expression, and PF can inhibit NP-induced *FASN* mRNA expression (Figs. 6c, 7c). In human cells, preadipocytes accumulated intracellular lipids after the induction of differentiation. NP alone could not increase lipid accumulation (Fig. 5c). The commercial PADM used in this study contains dexamethasone 1 μM with insulin 7.5 μg/ml, and dexamethasone combined with insulin dose-dependently upregulated adipogenesis and fat accumulation in cultured adipocytes [55]; therefore, the NP effect on adipogenic activity was masked by the optimized differentiation medium. PF decreased while NP increased PF-inhibited (PF at 1.5 or 2 μM) lipid accumulation (Fig. 5c). These results indicate that: 1) PF decreases PPARγ mRNA and protein expression and increases PPARα protein (Fig. 7a, d, e); 2) NP increases PPARγ mRNA and protein expression (Fig. 7a, d); and 3) depending on the dose, NP and PF have mutual antagonistic capabilities.

PPARα is a ligand-activated nuclear transcription factor that regulates lipid catabolism and inflammation. Increasing fatty acid oxidation by PPARα activation can lower circulating triglyceride levels, decrease liver and muscle steatosis, and reduce adiposity, which improves insulin sensitivity [56–58]. Morton et al. [15] demonstrated that 11β-HSD1-deficient mice exhibited increased PPARα expression in their livers to increase lipid catabolism, reduce intracellular glucocorticoid concentrations, and increase hepatic insulin sensitivity. In the rat model in this study, developmental exposure to NP significantly decreased the protein expression of PPARα in adipose tissues (Fig. 3b), and PF significantly alleviated NP-inhibited PPARα protein expression in adipose tissues (Fig. 3b) and adrenal glands (Fig. 4d). In human preadipocytes, P exposure to NP significantly decreased PPARα protein expression, and PF alleviated the NP-inhibited PPARα protein expression (*p* < 0.05, Fig. 6e). The results from both the human cell in vitro model and the rat in vivo model indicate that NP-induced adiposity can be alleviated or prevented by PF administration via increased PPARα protein expression and subsequently increased lipid catabolism. They also show that the rodent model is an adequate model to mimic NP’s effects and treatment in human cells. NP did not affect *PPARα* mRNA expression with either P or C exposure in human preadipocytes (Figs. 6b, 7b), possibly because NP may affect PPARα expression at the translation stage in human preadipocytes.

Bost et al. [59] proposed that transient activation of the MEK/MAPKs signaling pathway is required for the differentiation of preadipocytes. Additionally, the authors demonstrated that wild-type mice had more adipocytes and increased adiposity compared to ERK1^−^/^−^ mice [60]. Furthermore, the differentiation of preadipocytes isolated from ERK1^−^/^−^ mice was impaired [60]. PPARγ is expressed after MEK/MAPK activation in preadipocytes [61]. Adipocyte lipolysis is catalyzed by adipose triglyceride lipase and hormone-sensitive lipase, leading to hydrolysis of triglycerides to glycerol and free fatty acids [62]. Hormone-sensitive lipase is considered the rate-limiting enzyme in lipolysis [63], and its activity is mainly driven by phosphorylation of a serine residue through cAMP-dependent protein kinase A (PKA) [64]. This study shows that PF can alleviate NP-induced adipogenesis through the PPAR system; however, the associated mechanism is not clear. We will further explore the mechanisms of NP and PF in adipogenesis through MEK/MAPK, PKA, or other pathways.

We found that developmental exposure to NP results in hyperadrenalism in adulthood in our rat model. However, whether this phenomenon in rats reflects human conditions remains questionable. The most ethical approach would be to examine the effects of NP using in vitro models with human progenitor cells. Adipose tissue is one of the organs affected by NP; therefore, preadipocytes are a logical choice for studying the effects of NP. Priming human preadipocytes with NP for a short period of time (12 h, P exposure) results in decreased PPARα protein expression after 6 days of culture in differentiated adipocytes (Fig. 6e). These results are similar to the results from adipose tissues in the rat in vivo model (Fig. 3b). If human preadipocytes are continuously exposed to NP (C exposure, Fig. 7), the PPARγ and FASN response patterns are very similar between P (Fig. 6) and C exposure. These observations suggest the following 3 considerations. First, short-term NP exposure during critical developmental periods results in long-term consequences. Second, NP exposure results in adiposity. The 3rd consideration is whether the rat model can adequately reflect NP’s impact on human cells. Table 2 shows a compilation of data from a previous report [17] and from this study. The rat model included rat pups exposed to NP during the developmental period in an in vivo system. The human model included human preadipocytes exposed to NP before differentiation (P exposure) in an in vitro system. Generally, PPARα has lipolysis functions, while PPARγ has lipogenesis functions. The data show a trend of decreasing PPARα expression at the protein level with NP exposure, and PF can antagonize NP’s effects in both the rat and human models. NP increased PPARγ mRNA expression but not protein expression, and PF could significantly antagonize NP’s effects at both the mRNA and protein levels. This phenomenon is evident in both the rat and human models. NP had no significant effect on FASN mRNA and protein expression levels, whereas PF significantly decreased FASN mRNA expression but not protein expression in the rat model. In the human model, NP increased FASN mRNA and protein expression, although not significantly, at the protein level. PF appeared to decrease NP’s effects at both the mRNA and protein levels. The comparison of the effects of NP and PF shows similar responses between the human and rat models. Therefore, rats may serve as an appropriate experimental model for human study.

**Table 2:** Comparison of NP and PF effect on lipogenic attributes on rat adipose tissue and human preadipocyte

		mRNA			Protein		
		PPARα	PPARγ	FASN	PPARα	PPARγ	FASN
Rat	NP vs. control	n.s.^a^	*p* < 0.01^a^ increase	n.s.^a^	*p* < 0.05 decrease	n.s.	n.s.^a^
	NP + PF vs. NP	n.s.^a^	*p* < 0.01^a^ decrease	*p* < 0.05^a^ decrease	*p* < 0.01 increase	*p* < 0.01 decrease	n.s.^a^
Human	NP vs. control	n.s*.*	*p* < 0.05 increase	*p* < 0.05 increase	*p* < 0.01 decrease	n.s.	n.s.
	NP + PF vs. NP	*p* < 0.05 increase	*p* < 0.05 decrease	*p* < 0.05 decrease	*p* < 0.05 increase	*p* < 0.05 decrease	*p* < 0.05 decrease

*n.s* not significant; ^a^data from previous report (Chang et al. 2016) [17]

## Conclusions

NP exposure during developmental periods results in hyperadrenalism and adiposity. One of the mechanisms of NP’s action is mediated through cellular 11β-HSD1. Inhibition of 11β-HSD1 can alleviate or prevent the impact of NP on not only adrenal gland function but also on the intracellular Cushing’s state. NP’s mechanism of action appears to also be mediated through the cellular PPAR system. The results from the rat model adequately reflect the impact of NP and PF on human preadipocytes. These observations suggest that rats serve as an adequate experimental model to examine the impact of NP and possible treatments for humans. Therefore, these results offer hope for the treatment of pandemic Cushing’s syndrome, especially ENDR-induced Cushing’s syndrome.

## Abbreviations

11β-HSD1: 11β-hydroxysteroid dehydrogenase I
BPA: Bisphenol A
DDT: Dichlorodiphenyl-trichloroethane
DES: Diethylstilbestrol
EDC: Endocrine-disrupting chemical
ENDRs: Endocrine disruptors
ERK: Extracellular signal–regulated kinase
FASN: Fatty acid synthase
GAPDH: Glyceraldehyde-3-phosphate dehydrogenase
MAPK: Mitogen-activated protein kinase
NADPH: Nicotinamide adenine dinucleotide phosphate
NP: Nonylphenol
PADM: Preadipocyte differentiation medium
PAM: Preadipocyte medium
PF PF915275: 4′-cyano-biphenyl-4-sulfonic acid (6-amino-pyridin-2-yl)-amide
PKA: Protein kinase A
PPAR: Proliferator-activated receptor
PPARα: Proliferator-activated receptor α
PPARγ: Proliferator-activated receptor γ
RIA: Radioimmunoassay
StAR: Steroidogenic acute regulatory
